# Maternal Nativity and Residence in US Territories and Preterm Birth

**DOI:** 10.1001/jamanetworkopen.2026.3601

**Published:** 2026-03-26

**Authors:** Diana Montoya-Williams, Alejandra Barreto, Brielle Formanowski, Heather H. Burris, Sara C. Handley, Timothy Nelin, Scott A. Lorch, Robin Ortiz

**Affiliations:** 1 Division of Neonatology, Children’s Hospital of Philadelphia, Philadelphia, Pennsylvania; 2 Department of Pediatrics, Perelman School of Medicine, University of Pennsylvania, Philadelphia; 3 Children’s Hospital of Philadelphia PolicyLab, Philadelphia, Pennsylvania; 4 Leonard Davis Institute of Health Economics, University of Pennsylvania, Philadelphia, Pennsylvania; 5 Now with Yale University School of Medicine, New Haven, Connecticut; 6 Department of Obstetrics and Gynecology, Perelman School of Medicine, University of Pennsylvania, Philadelphia; 7 Department of Biostatistics, Epidemiology, and Informatics, Perelman School of Medicine, University of Pennsylvania, Philadelphia; 8 New York University Grossman School of Medicine, Departments of Pediatrics and Population Health; 9 New York University Langone Health Institute for Excellence in Health Equity

## Abstract

**Importance:**

US territory nativity and/or residence may be associated with health because it affects environmental exposures, insurance coverage, prenatal care, and other factors. Investigations of preterm birth in the US territories are limited.

**Objective:**

To assess the association between maternal territory status and preterm birth, as well as whether insurance type modified associations.

**Design, Setting, and Participants:**

Cross-sectional study of restricted-use birth certificates of in-hospital, singleton births in the US and territories from 2014 to 2023.

**Exposures:**

Maternal territory status was defined for births in territories where data were available (Guam, Northern Mariana Islands, Puerto Rico, and Virgin Islands) for the following groups: (1) those with territory nativity and residence; (2) those with territory nativity and mainland residence; (3) those with mainland nativity and territory residence; and (4) those with mainland nativity and residence (reference group). Insurance type (private, Medicaid, or other) was evaluated as well.

**Main Outcomes and Measures:**

Preterm birth (live birth before 37 weeks’ gestation).

**Results:**

Among 28 627 700 births, 465 291 (1.6%) had any maternal territory status (nativity or residence). This group had a mean (SD) age of 27.1 (6.0) years, and 297 593 (64.0%) had Medicaid insurance. The highest preterm birth rate was among those with territory nativity and residence (10.5%; 95% CI, 10.4%-10.7%); the lowest was among those with mainland nativity and residence (8.4%; 95% CI, 8.4%-8.5%). Individuals with territory nativity and residence had an adjusted relative risk (aRR) of 1.30 (95% CI, 1.29-1.32) for preterm birth compared with the reference group. There was significant interaction between territory residence and insurance. Compared with individuals with mainland residence and private insurance, those with territory residence and Medicaid had the highest preterm birth risk (aRR, 1.57; 95% CI, 1.55-1.59), followed by territory residence and private insurance (aRR, 1.42; 95% CI, 1.39-1.45).

**Conclusions and relevance:**

In this cross-sectional study, maternal territory nativity and residence were associated with preterm birth. Territory residence was associated with a higher risk of preterm birth, regardless of insurance type. Privately insured-individuals in US territories had a higher risk of preterm birth than Medicaid-insured individuals in the mainland. Given differential access to health care, health insurance, and other social exposures between territory and mainland populations, future work should explore causal effects and related policies that may improve birth outcomes in US territories.

## Key Points

**Question:**

What is the risk of preterm birth in US territories compared with the US mainland?

**Findings:**

In this cross-sectional study of US birth certificate data from 28 627 700 births, individuals who were born and resided in US territories had a 30% increased risk of preterm birth compared with those who were born and resided on the mainland, an association that was modified by insurance status and prenatal care.

**Meaning:**

These findings suggest that maternal territory status is associated with increased risk of preterm birth; this may be due to contextual factors that differ between the US territories and the mainland, such as policies impacting prenatal care access.

## Teaser

This cross-sectional study evaluates the association between maternal territory status and preterm birth, as well as whether insurance type modified associations.

## Introduction

Health disparities in the mainland US are often attributed to inequitable exposure to adverse social drivers of health (SDoH), such as lower socioeconomic status^1^ and racism.^2^ Significant health disparities have also been documented between people living in US territories and people living in the mainland US, primarily with respect to adult health outcomes like cancer, hypertension, and posthospitalization mortality.^3,4,5,6^ Higher rates of adverse birth outcomes like preterm birth (PTB) have been documented in some US territories, particularly among island-born compared with mainland-born Puerto Rican people.^7,8,9,10^ However, higher risk of PTB has also been documented among those born in Puerto Rico even when they live in the mainland US.^10,11^

Research on the health of people from the territories has been complicated by ongoing debates around how to categorize US territory–born people.^12^ While they are US citizens by birth,^13^ some have argued that people born in US territories should be categorized as foreign-born in epidemiologic research because if they live in the territories, health care and economic infrastructure are more similar to low to middle income countries than the mainland US.^14,15,16^ To our knowledge, no study has explored whether territory nativity vs territory residence have differential relationships with a population health outcome like PTB. There is also limited research on risk of PTB among those from US territories beyond Puerto Rico.^13^

This study aimed to assess the association between birthing person (hereafter maternal) territory nativity and residence with PTB using a novel definition of maternal territory status for perinatal health services research. We designated full maternal territory status for someone who was both born in a US territory (territory nativity) and lived in a US territory at the time of giving birth (territory residence). We hypothesized that maternal territory status would function as an adverse SDoH, with full territory status conferring higher PTB risk compared with counterparts born in or living in the mainland US during childbirth.

## Methods

### Conceptual Frameworks

This study’s design was informed by 3 conceptual frameworks. The first identifies the geography of where one lives as a determinant of health due to the built and psychosocial environments that make up the exposome.^17^ This exposome can include health-promoting policies that vary by county^17^ and state,^18^ as well as historical and contemporary social contexts, such as colonialism, which may be particularly important to health in US territories.^19^ The second framework highlights structural racism and discrimination as root causes of disparities in birth outcomes that manifest via inequitable exposures to low-quality public health and health care, housing, criminal justice, education, economic systems, and environment and climate change.^20^ The third framework was designed to understand health among Guamanians and highlights the roles of intergenerational trauma, forced socioeconomic dependency, and a lack of autonomy in the face of structural challenges as contributors to poor health among those living in this US territory.^21^ Using these frameworks, we posit that maternal ties to US territories are associated with disproportionate exposure to multilevel, structural discrimination via differential policies that increases risk of PTB, but the outcomes of the exposure vary by whether they were only born in a territory or have spent years living in one.

### Study Cohort

This cross-sectional study used restricted national birth certificate records from the National Center for Vital Statistics for all singleton, in-hospital, live births (98% of all births in the states^22^) for infants born between January 1, 2014, and December 31, 2023. Restricted-use vital statistics include county and state information and are accessible via data use agreements to protect confidentiality. The following were exclusion criteria: multiple births given the high risk of preterm delivery,^23^ infants with a gestational age (GA) less than 20 or more than 45 weeks, infants with a birth weight more than 5 SDs from the mean for GA, and maternal nativity or residence in a foreign country. Records with missing variables of interest (birth weight, GA, maternal nativity, and residence) were excluded (eFigure 1 in Supplement 1). We compared the demographics of those included in our study population with those excluded due to missing a variable of interest and found no differences (eTable 1 in Supplement 1). The Children's Hospital of Philadelphia institutional review board deemed this study exempt from ethics review and informed consent requirement because it was not considered human participant research. We followed the Strengthening the Reporting of Observational Studies in Epidemiology (STROBE) reporting guideline.

### Exposures

Maternal nativity is self-reported on US birth certificates. Individuals can select their birth country and postal state (eg, 50 states, US territories, Canadian Provinces, or foreign countries). Individuals who selected the US as their birth country or one of the 50 states of the US or DC as their birth state (including Hawaii and Alaska) were considered to have mainland nativity. US territory nativity was assigned if the maternal birth state was 1 of 4 US territories: Guam, Northern Mariana Islands, Puerto Rico, or the Virgin Islands. Although there are currently 10 other territories under US jurisdiction (ie, American Samoa and minor outlying islands mainly in the Pacific Ocean), birth certificates from these territories are not currently available and could not be analyzed in this study.^24^ Similarly, maternal residential country and state at the time of childbirth are self-reported on birth certificates, and the same categorization rules were applied. Infant place of birth was not considered separately as 98% of births occurred in the same place as maternal residence, making place of infant birth and maternal residence collinear. Maternal nativity and residence were combined into mutually exclusive categories to obtain territory status: (1) territory nativity and residence (born and living in the territories during childbirth); (2) territory nativity and mainland residence (born in the territories and living in the mainland during childbirth; (3) mainland nativity and territory residence (born in the mainland and living in the territories during childbirth; and (4) mainland nativity and residence (born and living in the mainland during childbirth).

### Covariates

The primary binary outcome was PTB (GA <37 weeks). The following maternal covariates known to be associated with PTB and reliably available on birth certificates were included: maternal age (≤19, 20-34, or ≥35 years); insurance type (private, Medicaid, or other); education level (<high school, some high school, high school diploma, or at least some college); presence of prenatal care (binary); any diabetes (diagnosed before or during pregnancy; binary); any hypertension (binary); and tobacco use (binary).^25,26,27,28,29^ Since congenital anomalies increase risk of PTB, models also adjusted for the presence of infant congenital anomalies (binary) using *International Classification of Diseases, Ninth and Tenth Revisions* codes from birth certificates.^30^ Missing values for these covariates were included as missing indicators^31^ because birth certificate data are often not missing at random.^32^ While race and ethnicity are proxies for critical SDoH, they were not included in analyses due to their inconsistent use in some territories, like Puerto Rico, where individuals often do not identify with a race.^33^ As our study focuses on territory status, the impact of race, ethnicity, and the closely related construct of colorism in US territories merits its own analysis and was beyond the scope of this study.

### Statistical Analysis

Descriptive analyses compared maternal and infant characteristics including PTB incidence by territory status with χ^2^ tests. We calculated the PTB rate for maternal residence (regardless of nativity) as well as maternal nativity (regardless of residence) in each of the 4 territories over the study period. Modified Poisson regression models then assessed PTB risk by maternal territory status with individuals born and living in the US mainland at childbirth as the reference group. Risk was estimated with and without adjustment for maternal sociodemographic factors, medical covariates, and birth year. Postestimation pairwise comparisons between different categories of territory status were estimated using Bonferroni-adjusted CIs.

Sensitivity analyses were performed on a subgroup with a minimum GA of 23 weeks to account for potential discrepancies in liveborn and fetal death designations for infants born at 21 to 22 weeks’ gestation in territories.^34^ Additional sensitivity analyses included assessing PTB risk by maternal territory residence only and maternal territory nativity only. To mitigate coronavirus pandemic-related effects, a fourth sensitivity analysis assessed PTB risk for births stratified into before and after the onset of the pandemic. The prepandemic period included births from January 2014 through March 2020, and the postpandemic period included births from April 2020 through December 2023.

Considering how differently Medicaid functions in US territories,^21^ we explored how insurance type might moderate the association between PTB and maternal territory status in secondary analyses. As insurance type at the time of the birthing person’s own birth cannot be ascertained from this dataset, we evaluated for an interaction between maternal residence at childbirth and insurance type with a Wald test. To determine whether outcomes were significantly different from one another, linear pairwise comparisons were conducted across 4 groups: Medicaid-insured mainland residents; privately insured mainland residents; Medicaid-insured territory residents; and privately insured territory residents. Bonferroni-adjusted CIs (and *P* value of .01) were used for the interaction models and pairwise comparisons.

All significance tests conducted were 2-tailed, with α = .05 unless Bonferroni correction was noted otherwise. Data were analyzed using Stata version 18 (StataCorp).

## Results

Among 28 627 700 births, 465 291 (1.6%) had any maternal territory status (nativity or residence) (Table) and 297 593 (64.0%) had Medicaid insurance. Among those with any territory status, slightly less than half had territory nativity and residence (229 291 births [49.3%]) and territory nativity and mainland residence (217 166 births [46.7%]), while just 4.0% (18 384 births) had mainland nativity and territory residence. Individuals with both territory residence and nativity were, on average, younger and had higher Medicaid insurance rates. Those with territory nativity but mainland residence had lower education levels. Individuals with mainland nativity and residence were most likely to report smoking during pregnancy. Receipt of any prenatal care was similar regardless of territory status.

**Table.** Demographics and Medical Characteristics of Birthing People and Infants (2014-2023) by Maternal Territory Status

Characteristic	Individuals, No. (%)				
	Overall (N = 28 627 700)	Maternal territory status^a^			
		Mainland-born, mainland resident (n = 28 162 859)	Territory-born, territory resident (n = 229 291)^b^	Territory-born, mainland resident (n = 217 166)^b^	Mainland-born, territory resident (n = 18 384)^b^
Age, y					
≤19	1 609 502 (5.6)	1 569 420 (5.6)	24 602 (10.7)	14 245 (6.6)	1235 (6.7)
20-34	22 521 902 (78.7)	22 158 980 (78.7)	180 058 (78.5)	168 456 (77.6)	14 408 (78.4)
≥35	4 496 296 (15.7)	4 434 459 (15.8)	24 631 (10.7)	34 465 (15.9)	2741 (14.9)
Education level					
<High school	314 983 (1.1)	307 424 (1.1)	3709 (1.6)	3619 (1.7)	231 (1.3)
Some high school	2 401 680 (8.4)	2 348 282 (8.3)	22 575 (9.9)	29 332 (13.5)	1491 (8.1)
High school diploma	7 493 643 (26.2)	7 347 006 (26.1)	69 376 (30.3)	72 271 (33.3)	4990 (27.1)
At least some college	17 993 609 (62.9)	17 739 657 (63.0)	132 590 (57.8)	109 899 (50.6)	11 463 (62.4)
Insurance type					
Medicaid	11 626 976 (40.61)	11 329 383 (40.2)	157 411 (68.7)	129 289 (59.5)	10 893 (59.3)
Private	14 904 098 (52.1)	14 764 983 (52.4)	64 251 (28.0)	70 073 (32.3)	4791 (26.1)
Other^c^	1 763 370 (6.2)	1 740 017 (6.2)	5251 (2.3)	15 848 (7.3)	2254 (12.3)
Any prenatal care	27 680 864 (96.7)	27 228 760 (96.7)	225 380 (98.3)	208 726 (96.1)	17 998 (97.9)
Tobacco use	2 762 295 (9.7)	2 750 318 (9.8)	2720 (1.2)	8982 (4.1)	275 (1.5)
Hypertension^d^	3 001 860 (10.5)	2 964 064 (10.5)	15 233 (6.6)	21 330 (9.8)	1233 (6.7)
Diabetes^d^	2 007 408 (7.0)	1 975 808 (7.0)	10 791 (4.7)	19 881 (9.2)	928 (5.1)
Presence of congenital anomalies in infant	89 914 (0.3)	88 105 (0.3)	1054 (0.5)	678 (0.3)	77 (0.4)
Preterm birth^e^	2 424 596 (8.5)	2 377 707 (8.4)	24 128 (10.5)	20 988 (9.7)	1773 (9.6)

^a^ All χ^2^analyses were significant at *P* < .001.

^b^ Data from the following territories were available and included: Guam, Puerto Rico, Northern Marianas, and Virgin Islands.

^c^ Included birthing people who used Indian Health Service, CHAMPUS/TRICARE, other government, and self pay as the payment source.

^d^ Condition was diagnosed before or during pregnancy.

^e^ Preterm birth is defined as a live birth with gestational length less than 37 weeks.

**Figure 1.**
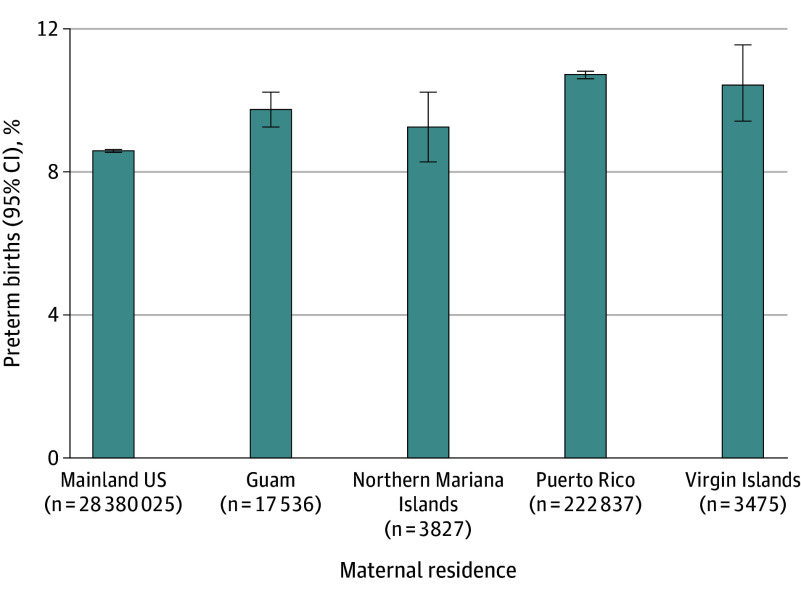
Bar Chart of Preterm US Births by Maternal Residence Preterm birth is defined as a gestational length of less than 37 weeks. All χ^2^ analyses were significant at *P* < .001.

Individuals with territory nativity and residence had the highest PTB rate (10.5%; 95% CI, 10.4%-10.7%) while individuals with mainland nativity and residence had the lowest (8.4%; 95% CI, 8.4%-8.5%). With respect to residence regardless of nativity, Puerto Rican residents had the highest PTB rate (10.6%; 95% CI, 10.4%-10.7%) and Northern Mariana Islands residents had the lowest (9.1%; 95% CI, 8.2%-10.1%). However, all territory residents had higher PTB rates than those living in the US mainland (Figure 1). When looking at maternal nativity alone, a similar pattern emerged except that those born in the US Virgin Islands had the highest rate of PTB (11.3%; 95% CI, 10.7%-11.8%) (eFigure 2 in Supplement 1).

**Figure 2.**
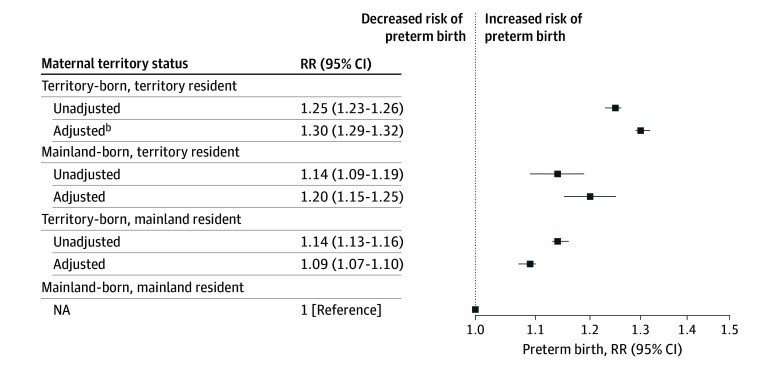
Forest Plot of Relative Risk (RR) of Preterm Birth by Maternal Territorial Status Preterm birth is defined as a gestational length less than 37 weeks. Models adjusted for maternal age, education, insurance, any prenatal care, tobacco use, hypertension, diabetes, congenital anomalies, and birth year.

After adjusting for covariates, individuals with any territory status had a higher relative risk of PTB compared with those with mainland nativity and residence (Figure 2). Individuals with territory nativity and residence had the most elevated risk (adjusted relative risk [aRR], 1.30; 95% CI, 1.29-1.32), followed by individuals with mainland nativity and territory residence (aRR, 1.20; 95% CI, 1.15-1.25) and individuals with territory nativity and mainland residence (aRR, 1.09; 95% CI, 1.07-1.10). When comparing individuals with territory residence, pairwise comparisons using Bonferroni-corrected CIs demonstrated that those with mainland nativity had significantly lower PTB risk than those with territory nativity (aRR, 0.92; 95% CI, 0.87-0.97). Among individuals with territory nativity, mainland residence was also associated with lower PTB risk (aRR, 0.83; 95% CI, 0.82-0.85) than territory residence. Sensitivity analyses considering maternal nativity and residence individually rather than in combination showed that territory exposure was still associated with higher PTB risk compared with mainland birth or residence (eTable 2 in Supplement 1). These associations were unchanged when births at less than 23 weeks’ gestation were excluded (eTable 3 in Supplement 1) and when only considering births before the onset of the pandemic in March 2020 (eTable 4 in Supplement 1). Following the onset of the pandemic, the risk of PTB remained significantly higher for individuals in all territory status groups compared with those with mainland nativity and residence, except among those with mainland nativity and territory residence (eTable 5 in Supplement 1).

**Figure 3.**
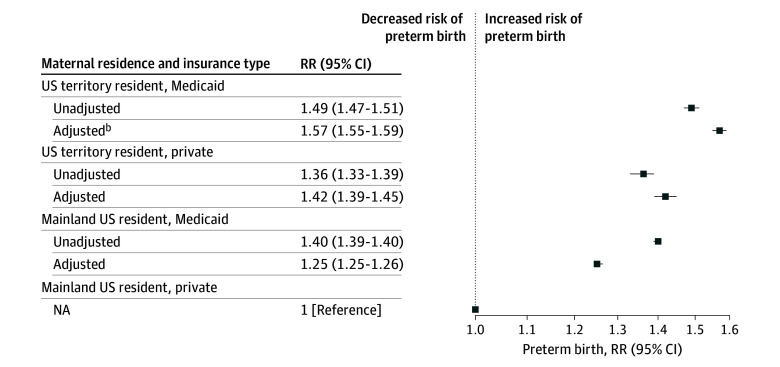
Forest Plot of Relative Risk (RR) of Preterm Birth by Maternal Residence and Insurance Type Linear combination testing of pairwise comparisons demonstrated that, in adjusted models, all effect estimates were significantly different (*P* < .01). Preterm birth is defined as a gestational length less than 37 weeks. Models adjusted for maternal age, education, insurance, any prenatal care, tobacco use, hypertension, diabetes, congenital anomalies, and birth year.

We detected a significant interaction between maternal territory residence and insurance. Compared with privately insured individuals with mainland residence, territory residence was associated with higher PTB risk regardless of their insurance coverage: (aRR for Medicaid-insured territory residents, 1.57; 95% CI, 1.55-1.59 and aRR for privately insured territory residents, 1.42; 95% CI, 1.39-1.45; *P* < .001) (Figure 3). Pairwise comparison testing demonstrated that these estimates were significantly different from each other. For instance, among individuals with territory residence, Medicaid (vs private) insurance was associated with higher PTB risk (aRR, 1.10; 95% CI, 1.07-1.14). Among individuals with Medicaid insurance, territory (vs mainland) residence was associated with higher PTB risk (aRR, 1.25; 95% CI, 1.23-1.28). Privately insured individuals with territory residence had a higher risk of PTB than Medicaid-insured individuals living in the mainland (aRR, 1.13; 95% CI, 1.10-1.17).

Given the close association between insurance coverage and prenatal care visits,^35^ a second post hoc exploratory interaction model tested for and found a significant interaction between maternal residence and any prenatal care (Wald test *P* value <.001). eTable 6 in Supplement 1 summarizes the relative risks of PTB for individuals by their interacted territory residence and prenatal care status.

## Discussion

In this national cross-sectional study, both US territory nativity and residence were associated with higher PTB risk, with the highest risk among those with both territory nativity and residence, followed by territory residents regardless of nativity. Prior research on PTB disparities associated with US territories has focused on Puerto Rico, reporting mixed results. This may be due to variation in how the exposure of territory status has been previously defined. For instance, several studies have examined Puerto Rican maternal nativity and its impact on PTB, with some finding no significant differences in birth cohorts from the 1990s^36^ and others finding increased risk of PTB for island-born Puerto Ricans^11^ more recently. A potentially different picture emerges from data looking at maternal residence in Puerto Rico at the time of childbirth compared with nativity. For instance, in 2003, the US Centers for Disease Control and Prevention (CDC) reported that PTB rates were higher among individuals giving birth on the island of Puerto Rico than for Puerto Rican people giving birth in the US mainland.^7^

Our novel approach of combining maternal territory nativity and residence may help reconcile prior mixed findings. Nativity has been posited to serve as a proxy for drivers of health that vary across the lifespan in multiple domains. On an interpersonal level, nativity may serve as a proxy for cultural practices that impact individual health behaviors.^37^ On a structural level, nativity may serve as a proxy for differential access to health-promoting resources like health insurance^37^ or can be used to study the impact of enclaves, ethnically and language concordant neighborhoods that offer social support and buffer socioeconomic deprivation.^38^ However, when considering associations of nativity with PTB, the birthing parent’s own birth is often a few decades removed from the outcome of PTB. Therefore, the addition of residence may help account for exposures more proximal to the PTB outcome.

This new intersectional variable of both maternal nativity and residence may help elucidate birth disparities experienced in US territories by better serving as a proxy for more proximal drivers of perinatal health. The highest risk we found that was created by intersecting both nativity and residence may help identify adverse structural drivers of health disproportionately faced by people in and from the US territories compared with the mainland that may be contributing to higher PTB risk. For instance, we found that health insurance appeared to modify the association between territory status and PTB, with the highest risks of PTB seen among those with Medicaid insurance and territory residence. Surprisingly, however, even those with private insurance and territory residence had higher PTB risk than those with Medicaid insurance and mainland residence. This suggests that higher PTB risk in the US territories cannot be attributed solely to eligibility for public insurance (a proxy for low socioeconomic status). Private insurance may be protective against PTB within territories as it is in the mainland, but other factors may be driving disparities that exist between territories and the mainland.

One important consideration is the political and economic marginalization experienced by US territories. Despite being US citizens who pay federal taxes, US territory residents lack representation in Congress and the electoral college during presidential elections.^39^ Disenfranchisement has been hypothesized to impact how policies related to health care delivery, reimbursement, and affordability are implemented.^39,40^ In our study, Medicaid was the payer for 69% of births with maternal territory nativity and residence, which is much higher than the 40% of births Medicaid covers in the mainland US.^41^ As a result, policies governing Medicaid and its coverage of prenatal care may help explain PTB disparities between the mainland and the territories. For instance, in the territories, federal reimbursement rates for Medicaid are lower^21^ and capped annually, compared with the currently uncapped federal funding rates with annual adjustments based on per capita income that states receive.^42^ Another hypothesis that could be explored is whether policies governing how Medicaid functions in the territories have downstream consequences on health care access and health outcomes of individuals with private insurance. In the mainland, for example, poor Medicaid reimbursement to hospitals has been postulated as a contributor to the creation of areas without maternity care^43^ and their associated poor maternal and infant health outcomes, even for privately insured populations.^44^

In addition to the combination of maternal nativity and residence, another strength of our study was the inclusion of PTB in territories that have been less well-explored in the literature. We found significant variation in population-level rates of PTB by territory residence. Interterritory comparisons of population health outcomes represent an understudied area but may shed light on how disparities between the mainland and territories emerge. Each US territory has a unique health care landscape, especially for perinatal care. For instance, Puerto Rico has a health care system comparable with that of some states, yet it lacks Medicaid-funded long-term care and nonemergency health transportation.^40^ The US Virgin Islands’ system has reduced service accessibility due to spread across separate islands.^40^ In Guam and the Northern Mariana Islands, Medicaid recipients may be incentivized to seek affordable care abroad due to the low availability of local clinicians that may reflect low rates of Medicaid reimbursement for care in those territories.^40^

Availability of neonatal intensive care units (NICU) also varies across the territories which may impact health care transfer patterns of pregnant people at high risk of PTB. For example, while Northern Mariana and the US Virgin Islands have NICUs that can provide intermediate care for very premature or critically ill infants (ie, level II and III NICUs),^45,46^ only Puerto Rico has a level IV NICU equipped to care for the most medically and surgically complex neonates.^47^ In contrast, Guam has no accredited NICU; high-risk pregnancies require transfers out of the territory as do high-risk neonates when possible.^48^ How availability and level of both obstetric and NICU care impacts PTB rates in the territories also merits future study.

### Limitations

Our study has limitations. US territories face significant challenges with data collection.^21,49^ The CDC’s public database does not include births in US territories and even the restricted-use datasets lack data from American Samoa, limiting research about this territory. It is unclear how this territory’s missing data would have changed the findings. In addition, the definition of a viable birth may vary among territories or hospitals based on local capabilities and resources. Differential policy classifications of pregnancy outcomes as fetal deaths vs live PTBs across territories could lead to undercounting of PTBs in the territories and thus possibly bias findings toward the null. However, sensitivity analyses using a higher gestational age for inclusion did not yield different results (eTable 2 in Supplement 1). Third, though length of residence in a new area may impact migrants’ birth outcomes,^50,51^ birth certificate limitations precluded our ability to assess length of residence among those giving birth in a place different from where they were born.

## Conclusions

In this cross-sectional study of over 28 million US births, individuals who were born in and resided in the US territories faced an increased risk of PTB than individuals who were born and resided in the US mainland. Furthermore, insurance type and presence of prenatal care emerged as important moderators of the association between maternal territory status and PTB. Our approach of categorizing individuals by combined maternal nativity and residence allows for an innovative approach that acknowledges the potential dynamic and intersectional impact of maternal nativity and residence on risk of poor birth outcomes. Future studies can build on these findings to explore mediators and potential policy solutions to territory-related disparities.

## Notes

Supplement 1. eFigure 1. Flow Chart of Participants Included in Analysis eFigure 2. Preterm Birth Rates by Maternal Nativity eTable 1. Comparison of Demographics and Medical Characteristics of Birthing People and Infants Among the Analytic Samplea and Population of Interest eTable 2. Relative Risk (RR) of Preterm Birth by Maternal Nativity Alone or Residence Alone eTable 3. Sensitivity Analysis of the Relative Risk (RR) of Preterm Birth by Maternal Nativity and Excluding Births Less Than 23 Weeks of Gestation eTable 4. Sensitivity Analysis of the Relative Risk of Preterm Birth by Nativity and Residence in the Mainland US and US Territories Restricted to Years Before the COVID-19 Pandemic (January 2014 to March 2020) eTable 5. Sensitivity Analysis of the Relative Risk of Preterm Birth by Nativity and Residence in the Mainland US and US Territories Restricted to Years After the COVID-19 Pandemic (April 2020 to December 2023) eTable 6. Relative Risk (RR) of Preterm Birth by Maternal Residence and Presence of Prenatal Care

Supplement 2. Data Sharing Statement

## References

[1] Telfair J, Shelton TL. Educational attainment as a social determinant of health. N C Med J. 2012;73(5):358-365. doi:10.18043/ncm.73.5.358 23189418

[2] Ramaswamy M, Kelly PJ. Institutional racism as a critical social determinant of health. Public Health Nurs. 2015;32(4):285-286. doi:10.1111/phn.12212 26199054

[3] Hernandez BY, Bordallo RA, Green MD, Haddock RL. Cancer in Guam and Hawaii: a comparison of two U.S. island populations. Cancer Epidemiol. 2017;50(Pt B):199-206. doi:10.1016/j.canep.2017.08.005 29120826 PMC5806134

[4] Ho GYF, Figueroa-Vallés NR, De La Torre-Feliciano T, . Cancer disparities between mainland and island Puerto Ricans. Rev Panam Salud Publica. 2009;25(5):394-400. doi:10.1590/S1020-49892009000500003 19695128

[5] Garcia C, Garcia MA, Ailshire JA. Sociocultural variability in the Latino population: age patterns and differences in morbidity among older US adults. Demogr Res. 2018;38:1605-1618. doi:10.4054/DemRes.2018.38.52 30416374 PMC6223319

[6] Nunez-Smith M, Bradley EH, Herrin J, . Quality of care in the US territories. Arch Intern Med. 2011;171(17):1528-1540. doi:10.1001/archinternmed.2011.284 21709184 PMC3251926

[7] Centers for Disease Control and Prevention (CDC). Infant health among Puerto Ricans–Puerto Rico and U.S. mainland, 1989-2000. MMWR Morb Mortal Wkly Rep. 2003;52(42):1012-1016. 14574275

[8] Landale NS, Gorman BK, Oropesa RS. Selective migration and infant mortality among Puerto Ricans. Matern Child Health J. 2006;10(4):351-360. doi:10.1007/s10995-006-0072-4 16721666

[9] Almeida J, Mulready-Ward C, Bettegowda VR, Ahluwalia IB. Racial/Ethnic and nativity differences in birth outcomes among mothers in New York City: the role of social ties and social support. Matern Child Health J. 2014;18(1):90-100. doi:10.1007/s10995-013-1238-5 23435918 PMC10999902

[10] Huang X, Lee K, Wang MC, . Maternal nativity and preterm birth. JAMA Pediatr. 2024;178(1):65-72. doi:10.1001/jamapediatrics.2023.4907 37955913 PMC10644246

[11] Britton ML. Boricuas, barrios and birth outcomes: residential segregation and preterm birth among Puerto Ricans in the United States. Centro Journal. 2015;27(1):70-99.

[12] Montoya-Williams D, Barreto A, Fuentes-Afflick E, Collins JW Jr. Nativity and perinatal outcome disparities in the United States: beyond the immigrant paradox. Semin Perinatol. 2022;46(8):151658. doi:10.1016/j.semperi.2022.151658 36137831 PMC10016119

[13] Barreto A, Formanowski B, Peña MM, . Preterm birth risk and maternal nativity, ethnicity, and race. JAMA Netw Open. 2024;7(3):e243194. doi:10.1001/jamanetworkopen.2024.3194 38512251 PMC10958237

[14] Goel MS, Wee CC, McCarthy EP, Davis RB, Ngo-Metzger Q, Phillips RS. Racial and ethnic disparities in cancer screening: the importance of foreign birth as a barrier to care. J Gen Intern Med. 2003;18(12):1028-1035. doi:10.1111/j.1525-1497.2003.20807.x 14687262 PMC1494963

[15] Choi D, Narayan KMV, Patel SA. Disparities in diabetes between US-born and foreign-born population: using three diabetes indicators. Biodemography Soc Biol. 2022;67(1):16-27. doi:10.1080/19485565.2021.2016368 35466846 PMC9039242

[16] Koya DL, Egede LE. Association between length of residence and cardiovascular disease risk factors among an ethnically diverse group of United States immigrants. J Gen Intern Med. 2007;22(6):841-846. doi:10.1007/s11606-007-0163-y 17503110 PMC2219871

[17] Johnson K, Oruganti N, Cilenti D, Wiesman J, Jensen T, Hassmiller K. Local public health strategies for addressing social determinants of health-analysis of recent community health improvement plans. J Public Health Manag Pract. 2024;30(6):823-831. doi:10.1097/PHH.0000000000001938 39255502

[18] Chang L, Puls HT, Monuteaux MC, Colvin JD, Chung PJ, Lee LK. State social expenditures and preterm birth and low birth weight in the US. JAMA Pediatr. 2024;178(12):1345-1353. doi:10.1001/jamapediatrics.2024.4267 39401047 PMC11581729

[19] Pérez Ramos JG, Garriga-López A, Rodríguez-Díaz CE. How is colonialism a sociostructural determinant of health in Puerto Rico? AMA J Ethics. 2022;24(4):E305-E312. doi:10.1001/amajethics.2022.305 35405057

[20] Sonderlund AL, Charifson M, Ortiz R, Khan M, Schoenthaler A, Williams NJ. A comprehensive framework for operationalizing structural racism in health research: the association between mass incarceration of Black people in the US and adverse birth outcomes. SSM-Popul Health. 2022;19:101225. doi:10.1016/j.ssmph.2022.101225 PMC9513165 36177482

[21] Diaz TP, Ka’opua LSI, Nakaoka S. Island nation, US territory and contested space: territorial status as a social determinant of indigenous health in Guam. Br J Soc Work. 2020;50(4):1069-1088. doi:10.1093/bjsw/bcz097 32753769 PMC7402591

[22] MacDorman MF, Declercq E. Trends and state variations in out-of-hospital births in the United States, 2004-2017. Birth. 2019;46(2):279-288. doi:10.1111/birt.12411 30537156 PMC6642827

[23] Goldenberg RL, Culhane JF, Iams JD, Romero R. Epidemiology and causes of preterm birth. Lancet. 2008;371(9606):75-84. doi:10.1016/S0140-6736(08)60074-4 18177778 PMC7134569

[24] Osterman MJK, Hamilton BE, Martin JA, Driscoll AK, Valenzuela CP. Births: final data for 2021. Natl Vital Stat Rep. 2023;72(1):1-53. 36723449

[25] Fuchs F, Monet B, Ducruet T, Chaillet N, Audibert F. Effect of maternal age on the risk of preterm birth: a large cohort study. PLoS One. 2018;13(1):e0191002. doi:10.1371/journal.pone.0191002 29385154 PMC5791955

[26] Shin D, Song WO. Influence of the adequacy of the prenatal care utilization index on small-for-gestational-age infants and preterm births in the United States. J Clin Med. 2019;8(6):838. doi:10.3390/jcm8060838 31212823 PMC6616923

[27] Ion R, Bernal AL. Smoking and preterm birth. Reprod Sci. 2015;22(8):918-926. doi:10.1177/1933719114556486 25394641

[28] Berger H, Melamed N, Davis BM, . Impact of diabetes, obesity and hypertension on preterm birth: population-based study. PLoS One. 2020;15(3):e0228743. doi:10.1371/journal.pone.0228743 32210434 PMC7094836

[29] Thoma ME, Drew LB, Hirai AH, Kim TY, Fenelon A, Shenassa ED. Black-White disparities in preterm birth: geographic, social, and health determinants. Am J Prev Med. 2019;57(5):675-686. doi:10.1016/j.amepre.2019.07.007 31561920

[30] Phibbs CS, Baker LC, Caughey AB, Danielsen B, Schmitt SK, Phibbs RH. Level and volume of neonatal intensive care and mortality in very-low-birth-weight infants. N Engl J Med. 2007;356(21):2165-2175. doi:10.1056/NEJMsa065029 17522400

[31] Groenwold RHH, White IR, Donders ART, Carpenter JR, Altman DG, Moons KGM. Missing covariate data in clinical research: when and when not to use the missing-indicator method for analysis. CMAJ. 2012;184(11):1265-1269. doi:10.1503/cmaj.110977 22371511 PMC3414599

[32] Gould JB, Chavez G, Marks AR, Liu H. Incomplete birth certificates: a risk marker for infant mortality. Am J Public Health. 2002;92(1):79-81. doi:10.2105/AJPH.92.1.79 11772766 PMC1447393

[33] Godreau I, Bonilla Y. Nonsovereign racecraft: how colonialism, debt, and disaster are transforming Puerto Rican racial subjectivities. Am Anthropol. 2021;123(3):509-525. doi:10.1111/aman.13601

[34] Patterson JK, Aziz A, Bauserman MS, McClure EM, Goldenberg RL, Bose CL. Challenges in classification and assignment of causes of stillbirths in low- and lower middle-income countries. Semin Perinatol. 2019;43(5):308-314. doi:10.1053/j.semperi.2019.03.021 30981473 PMC7894980

[35] Palmer M. Preconception subsidized insurance: prenatal care and birth outcomes by race/ethnicity. Health Econ. 2020;29(9):1013-1030. doi:10.1002/hec.4116 32529714

[36] Engel T, Alexander GR, Leland NL. Pregnancy outcomes of U.S.-born Puerto Ricans: the role of maternal nativity status. Am J Prev Med. 1995;11(1):34-39. doi:10.1016/S0749-3797(18)30498-7 7748584

[37] Marouf FE, Perreira KM, Pham H. Adding nativity, citizenship, and immigration status to health monitoring and survey data. Am J Public Health. 2025;115(1):75-82. doi:10.2105/AJPH.2024.307867 39631085 PMC11628710

[38] Viruell-Fuentes EA, Morenoff JD, Williams DR, House JS. Contextualizing nativity status, Latino social ties, and ethnic enclaves: an examination of the ‘immigrant social ties hypothesis’. Ethn Health. 2013;18(6):586-609. doi:10.1080/13557858.2013.814763 23947776 PMC4176765

[39] Borgonia J. Voting rights denied by residency: enfranchising millions of U.S.Citizens in U.S. territories. Seattle J Soc Justice. 2021;19(3):841-884.

[40] Gutierrez N. Understanding health care disparities in the US territories. Arch Intern Med. 2011;171(17):1579-1581. doi:10.1001/archinternmed.2011.305 21709183

[41] Center for Medicaid and CHIP Services. 2024 Medicaid and CHIP beneficiaries at a glance:maternal health. May 2024. Accessed February 13, 2026. https://www.medicaid.gov/medicaid/benefits/downloads/2024-maternal-health-at-a-glance.pdf

[42] Stolyar L, Rudowitz R. Medicaid financing and the U.S. territories: implications of the Build Back Better Act. KFF. February 9, 2022. Accessed July 2, 2024. https://www.kff.org/policy-watch/medicaid-financing-and-u-s-territories-implications-build-back-better-act/

[43] Sonenberg A, Mason DJ. Maternity care deserts in the US. JAMA Health Forum. 2023;4(1):e225541. doi:10.1001/jamahealthforum.2022.5541 36633853

[44] State-level prevalence of maternity care deserts: association with healthcare access, utilization, and outcomes among Medicaid recipients. AJPM Focus. 2025;4(5):100362. doi:10.1016/j.focus.2025.100362 PMC12305214 40735223

[45] U.S. Department of Health and Human Services. III.A.1. Program Overview—Northern Mariana Islands—2024. 2024. Accessed February 13, 2026. https://mchb.tvisdata.hrsa.gov/Narratives/ExecutiveSummary/726aa275-170d-4d3d-83ce-0a7d432e7602

[46] CareerMD. Governor Juan F. Luis Hospital and Medical Center. Accessed June 19, 2025. https://app.careermd.com/physicians/careerfairs/employersnapshot.aspx?pid=244447075&utm

[47] University of Puerto Rico School of Medicine. Department of pediatrics, neonatology section. Neonatology. Accessed June 19, 2025. https://md.rcm.upr.edu/pediatrics/neonatology/

[48] Kime P. The military on Guam has no intensive care unit for newborns in critical condition. Some don’t make it. Military.com. June 18, 2024. Accessed August 2, 2024. https://www.military.com/daily-news/2024/06/18/guam-lacks-newborn-intensive-care-unit-potentially-putting-hundreds-of-babies-born-troops-each-year.html

[49] Marie McSorley AM, Wheatley A, Pagán JA. A call to increase health data availability in US territories-not too small to count. JAMA Health Forum. 2023;4(9):e233088. doi:10.1001/jamahealthforum.2023.3088 37738063

[50] Crump C, Lipsky S, Mueller BA. Adverse birth outcomes among Mexican-Americans: are US-born women at greater risk than Mexico-born women? Ethn Health. 1999;4(1-2):29-34. doi:10.1080/13557859998164 10887459

[51] Elsayed A, Amutah-Onukagha NN, Navin L, Gittens-Williams L, Janevic T. Impact of immigration and duration of residence in US on length of gestation among black women in Newark, New Jersey. J Immigr Minor Health. 2019;21(5):1095-1101. doi:10.1007/s10903-018-0813-7 30171430 PMC9994288

