# Supplementary material for: Maternal Nativity and Residence in US Territories and Preterm Birth
Source: JAMA Netw Open. 2026 Mar 26;9(3):e263601. doi: 10.1001/jamanetworkopen.2026.3601 (PMC13022733; doi:10.1001/jamanetworkopen.2026.3601)
Supplement: Supplement 1. — eFigure 1. Flow Chart of Participants Included in Analysis eFigure 2. Preterm Birth Rates by Maternal Nativity eTable 1. Comparison of Demographics and Medical Characteristics of Birthing People and Infants Among the Analytic Samplea and Population of Interest eTable 2. Relative Risk (RR) of Preterm Birth by Maternal Nativity Alone or Residence Alone eTable 3. Sensitivity Analysis of the Relative Risk (RR) of Preterm Birth by Maternal Nativity and Excluding Births Less Than 23 Weeks of Gestation eTable 4. Sensitivity Analysis of the Relative Risk of Preterm Birth by Nativity and Residence in the Mainland US and US Territories Restricted to Years Before the COVID-19 Pandemic (January 2014 to March 2020) eTable 5. Sensitivity Analysis of the Relative Risk of Preterm Birth by Nativity and Residence in the Mainland US and US Territories Restricted to Years After the COVID-19 Pandemic (April 2020 to December 2023) eTable 6. Relative Risk (RR) of Preterm Birth by Maternal Residence and Presence of Prenatal Care [file jamanetwopen-e263601-s001.pdf]

Supplemental Online Content

Montoya-Williams D, Barreto A, Formanowski B, et al. Maternal nativity and residence in US territories and preterm birth. *JAMA Netw Open*. 2026;9(3):e263601. doi:10.1001/jamanetworkopen.2026.3601

eFigure 1. Flow Chart of Participants Included in Analysis
eFigure 2. Preterm Birth Rates by Maternal Nativity
eTable 1. Comparison of Demographics and Medical Characteristics of Birthing People and Infants Among the Analytic Samplea and Population of Interest
eTable 2. Relative Risk (RR) of Preterm Birth by Maternal Nativity Alone or Residence Alone
eTable 3. Sensitivity Analysis of the Relative Risk (RR) of Preterm Birth by Maternal Nativity and Excluding Births Less Than 23 Weeks of Gestation
eTable 4. Sensitivity Analysis of the Relative Risk of Preterm Birth by Nativity and Residence in the Mainland US and US Territories Restricted to Years Before the COVID-19 Pandemic (January 2014 to March 2020)
eTable 5. Sensitivity Analysis of the Relative Risk of Preterm Birth by Nativity and Residence in the Mainland US and US Territories Restricted to Years After the COVID-19 Pandemic (April 2020 to December 2023)
eTable 6. Relative Risk (RR) of Preterm Birth by Maternal Residence and Presence of Prenatal Care

This supplemental material has been provided by the authors to give readers additional information about their work.

© 2026 Montoya-Williams D et al. *JAMA Network Open*.

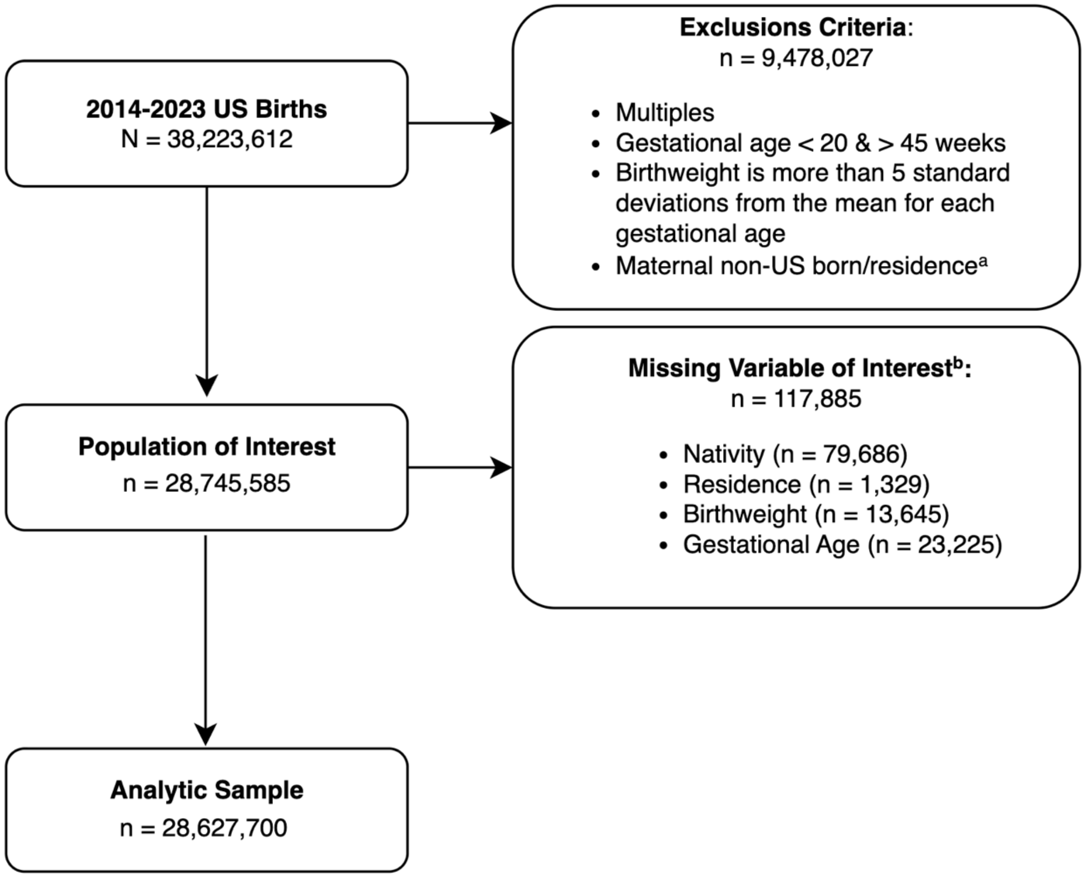

eFigure 1. Flow Chart of Participants Included in Analysis

Caption: [a]Excludes individuals with American Samoan nativity and residence due to unavailable data from the National Vital Statistics Office; [b]Births may have more than one missing variable

© 2026 Montoya-Williams D et al. *JAMA Network Open*.

eFigure 2: Preterm Birth[a] Rates by Maternal Nativity

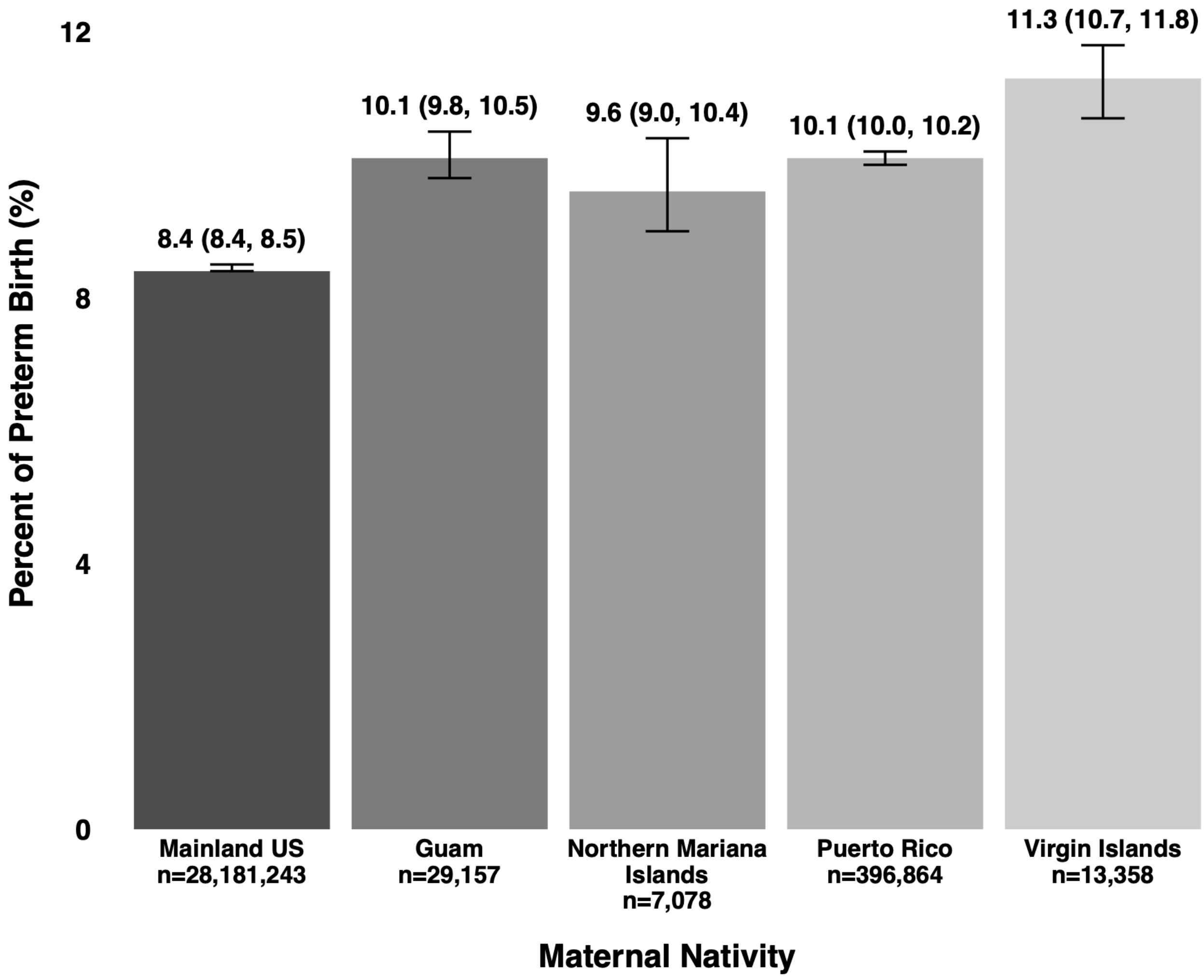

[a]Preterm birth is defined by a gestational length of <37 weeks
All χ^2 analysis were significant at $P < .001$.

© 2026 Montoya-Williams D et al. *JAMA Network Open*.

eTable 1. Comparison of Demographics and Medical Characteristics of Birthing People and Infants Among the Analytic Sample[a] and Population of Interest

	Analytic Sample<br>n = 28,627,700<br>No. (%)	Population of Interest<br>(Missing a Variable of Interest[b])<br>n = 28,745,585<br>No. (%)
Age, years		
≤19	1,609,502 (5.62)	1,616,495 (5.62)
20-34	22,521,902 (78.67)	22,610,377 (78.66)
≥35	4,496,296 (15.71)	4,518,713 (15.72)
Education Level		
Missing	423,785 (1.48)	448,551 (1.56)
< High School	314,983 (1.10)	320,370 (1.11)
Some High School	2,401,680 (8.39)	2,414,964 (8.40)
High School Diploma	7,493,643 (26.18)	7,522,100 (26.17)
At Least Some College	17,993,609 (62.85)	18,039,600 (62.76)
Insurance Type		
Missing	333,256 (1.16)	347,425 (1.21)
Medicaid	11,626,976 (40.61)	11,680,700 (40.63)
Private	14,904,098 (52.06)	14,939,939 (51.97)
Other[c]	1,763,370 (6.16)	1,777,521 (6.18)
Any Prenatal Care		
Missing	482,122 (1.68)	500,713 (1.74)
No	464,714 (1.62)	481,905 (1.68)
Yes	27,680,864 (96.69)	27,762,967 (96.58)
Tobacco Use		
Missing	378,274 (1.32)	392,190 (1.36)
No	25,487,131 (89.03)	25,579,064 (88.98)
Yes	2,762,295 (9.65)	2,774,331 (9.65)
Hypertension[d]		
Missing	31,762 (0.11)	39,868 (0.14)
No	25,594,078 (89.40)	25,694,652 (89.39)
Yes	3,001,860 (10.49)	3,011,065 (10.47)
Diabetes[d]		
Missing	31,762 (0.11)	39,868 (0.14)
No	26,588,530 (92.88)	26,691,485 (92.85)
Yes	2,007,408 (7.01)	2,014,232 (7.01)
Birth Year		
2014	3,021,125 (10.55)	3,034,644 (10.56)
2015	3,007,395 (10.51)	3,020,217 (10.51)
2016	2,964,341 (10.35)	2,977,528 (10.36)
2017	2,896,195 (10.12)	2,906,947 (10.11)
2018	2,863,329 (10.00)	2,873,867 (10.00)
2019	2,836,499 (9.91)	2,847,917 (9.91)
2020	2,760,253 (9.64)	2,771,107 (9.64)
2021	2,818,392 (9.84)	2,830,063 (9.85)
2022	2,769,506 (9.67)	2,781,268 (9.68)
2023	2,690,665 (9.40)	2,702,027 (9.40)
Presence of congenital anomalies in infant		
Missing	193,732 (0.68)	202,025 (0.70)
No	28,344,054 (99.01)	28,452,947 (98.98)
Yes	89,914 (0.31)	90,613 (0.32)

[a]Data from the following territories were available and included: Guam, Puerto Rico, Northern Marianas, and Virgin Islands.; [b]Missing variables of interest include: birthing person nativity, residence, infant birthweight and

© 2026 Montoya-Williams D et al. *JAMA Network Open*.

gestational age; [c]Included birthing people who used Indian Health Service, CHAMPUS/TRICARE, other government, and self pay as the payment source; [d]Condition was diagnosed before or during pregnancy

© 2026 Montoya-Williams D et al. *JAMA Network Open*.

eTable 2. Relative Risk (RR) of Preterm Birth by Maternal Nativity Alone or Residence Alone

	RR (95% CI)	aRR[a](95% CI)
**Maternal Nativity**		
Mainland	Ref.	Ref.
Territories	1.20 (1.19,1.21)	1.19 (1.18,1.20)
**Maternal Residence**		
Mainland	Ref.	Ref.
Territories	1.24 (1.22,1.25)	1.29 (1.28,1.31)

[a]Models adjust for maternal age, education, insurance, any prenatal care, tobacco use, any hypertension and diabetes, congenital anomalies, and birth year.

© 2026 Montoya-Williams D et al. *JAMA Network Open*.

eTable 3. Sensitivity Analysis of the Relative Risk (RR) of Preterm Birth by Maternal Nativity and Excluding Births Less Than 23 Weeks of Gestation (n=28,603,233)

	RR (95% CI)	aRR[a] (95% CI)
**Maternal Nativity**		
Mainland	Ref.	Ref.
Territories	1.20 (1.19, 1.21)	1.20 (1.18,1.21)
**Maternal Residence**		
Mainland	Ref.	Ref.
Territories	1.25 (1.23,1.26)	1.30 (1.29,1.32)
**Maternal Territory Status**		
Mainland-born, mainland resident	Ref.	Ref.
Territory-born, territory resident	1.25 (1.24,1.27)	1.31 (1.30,1.33)
Territory-born, mainland resident	1.14 (1.12,1.15)	1.08 (1.07,1.10)
Mainland-born, territory resident	1.15 (1.10,1.20)	1.21 (1.16,1.26)

[a]Models adjust for maternal age, education, insurance, any prenatal care, tobacco use, any hypertension and diabetes, congenital anomalies, and birth year.

© 2026 Montoya-Williams D et al. *JAMA Network Open*.

eTable 4. Sensitivity Analysis of the Relative Risk of Preterm Birth by Nativity and Residence in the Mainland US and US Territories Restricted to Years Before the COVID-19 Pandemic (January 2014 to March 2020) (n=18,263,758)

	RR (95% CI)	aRR[a] (95% CI)
**Maternal Territory Status**		
Mainland-born, mainland resident	Ref.	Ref.
Territory-born, territory resident	1.26 (1.24,1.28)	1.33 (1.31,1.35)
Territory-born, mainland resident	1.15 (1.13,1.17)	1.09 (1.07,1.10)
Mainland-born, territory resident	1.19 (1.24,1.28)	1.25 (1.19,1.32)

[a]Models adjust for maternal age, education, insurance, any prenatal care, tobacco use, any hypertension and diabetes, congenital anomalies, and birth year.

© 2026 Montoya-Williams D et al. *JAMA Network Open*.

eTable 5. Sensitivity Analysis of the Relative Risk of Preterm Birth by Nativity and Residence in the Mainland US and US Territories Restricted to Years After the COVID-19 Pandemic (April 2020 to December 2023) (n=10,363,942)

	RR (95% CI)	aRR[a] (95% CI)
**Maternal Territory Status**		
Mainland-born, mainland resident	Ref.	Ref.
Territory-born, territory resident	1.22 (1.20,1.25)	1.20 (1.18,1.23)
Territory-born, mainland resident	1.14 (1.11,1.16)	1.08 (1.06,1.11)
Mainland-born, territory resident	1.05 (0.96,1.14)	0.98 (0.90,1.07)

[a]Models adjust for maternal age, education, insurance, any prenatal care, tobacco use, any hypertension and diabetes, congenital anomalies, and birth year.

© 2026 Montoya-Williams D et al. *JAMA Network Open*.

eTable 6. Relative Risk (RR) of Preterm Birth by Maternal Residence and Presence of Prenatal Care

	RR (95% CI)	aRR[a] (95% CI)
**Maternal Residence & Prenatal Care Status**		
Mainland resident, any prenatal care	Ref.	Ref.
Mainland resident, no prenatal care	3.21 (3.19,3.22)	2.83 (2.81,2.84)
Mainland resident, missing prenatal care data	2.06 (2.04,2.07)	1.92 (1.92,1.93)
Territory resident, any prenatal care	1.28 (1.26,1.29)	1.31 (1.30,1.33)
Territory residence, no prenatal care	3.52 (3.24,3.82)	3.14 (2.89,3.41)
Territory resident, missing prenatal care data	2.64 (2.46,2.83)	1.72 (1.60,1.86)

[a]Models adjust for maternal age, education, insurance, tobacco use, any hypertension and diabetes, congenital anomalies, and birth year. Bonferroni-corrected CI were used for these analyses.

© 2026 Montoya-Williams D et al. *JAMA Network Open*.
