# Supplementary material for: Maternal Nativity and Residence in US Territories and Preterm Birth
Source: JAMA Netw Open. 2026 Mar 26;9(3):e263601. doi: 10.1001/jamanetworkopen.2026.3601 (PMC13022733; doi:10.1001/jamanetworkopen.2026.3601)
Supplement: Supplement 2. — Data Sharing Statement [file jamanetwopen-e263601-s002.pdf]

Data Sharing Statement

Montoya-Williams. Maternal Nativity and Residence in US Territories and Preterm Birth. *JAMA Netw Open*. Published March 26, 2026. doi:10.1001/jamanetworkopen.2026.3601

Data
Data available: No

Additional Information
Explanation for why data not available: This study examined restricted used data from the National Vital Statistics Office and thus data must be requested from them through a Data Use Agreement. For more information about analytic code and data definitions, please email the corresponding author.
